# Room-Temperature Catalyst Enables Selective Acetone Sensing

**DOI:** 10.3390/ma14081839

**Published:** 2021-04-08

**Authors:** Ines C. Weber, Chang-ting Wang, Andreas T. Güntner

**Affiliations:** 1 Particle Technology Laboratory, Department of Mechanical and Process Engineering, ETH Zurich, CH-8092 Zurich, Switzerland; iweber@ethz.ch (I.C.W.); chwang@student.ethz.ch (C.-t.W.); 2 Department of Endocrinology, Diabetology, and Clinical Nutrition, University Hospital Zurich (USZ) and University of Zurich (UZH), CH-8091 Zurich, Switzerland

**Keywords:** nanotechnology, combustion synthesis, electronics, semiconductors, metal oxides, noble metals

## Abstract

Catalytic packed bed filters ahead of gas sensors can drastically improve their selectivity, a key challenge in medical, food and environmental applications. Yet, such filters require high operation temperatures (usually some hundreds °C) impeding their integration into low-power (e.g., battery-driven) devices. Here, we reveal room-temperature catalytic filters that facilitate highly selective acetone sensing, a breath marker for body fat burn monitoring. Varying the Pt content between 0–10 mol% during flame spray pyrolysis resulted in Al_2_O_3_ nanoparticles decorated with Pt/PtO_x_ clusters with predominantly 5–6 nm size, as revealed by X-ray diffraction and electron microscopy. Most importantly, Pt contents above 3 mol% removed up to 100 ppm methanol, isoprene and ethanol completely already at 40 °C and high relative humidity, while acetone was mostly preserved, as confirmed by mass spectrometry. When combined with an inexpensive, chemo-resistive sensor of flame-made Si/WO_3_, acetone was detected with high selectivity (≥225) over these interferants next to H_2_, CO, form-/acetaldehyde and 2-propanol. Such catalytic filters do not require additional heating anymore, and thus are attractive for integration into mobile health care devices to monitor, for instance, lifestyle changes in gyms, hospitals or at home.

## 1. Introduction

Chemical gas sensors are promising for the next generation of handheld devices for air [1] or food quality monitoring [2], medical breath analysis [3] and human detection (e.g., in search and rescue [4] or translational crime control [5]). Additional filters [6] can drastically improve their performance to meet the challenging selectivity requirements of these applications, such as the quantification of single analytes among >800 [7] compounds in breath or >250 [8] in indoor air. Particularly interesting are catalytic filters that can convert interferants completely and continuously to sensor-inert species, while target analytes remain unaffected. Such filters have been investigated to remove confounders like CO [9] and ethanol [10] in alkane detection and very recently enabled selective benzene detection [1].

Breath acetone is a metabolic marker for lipolysis, and is thus interesting for monitoring the effectiveness of lifestyle changes (e.g., fasting [11], ketogenic dieting [12] and exercise [13]) or the treatment of metabolic diseases [14]. However, current acetone sensors lack sufficient selectivity to interferants. For instance, Co-doped ZnO nanofibers [15] and TiO_2_/WO_3_ nanocrystals [16] respond to H_2_ that forms in the intestinal tract after food intake and can reach significantly higher concentrations (>50 ppm) than breath acetone (e.g., 700–1000 ppb during exercise [17]). Al-ZnO [18] and Si/WO_3_ [19] are interfered by isoprene that spikes during physical activity [17]. Finally, SnO_2_, with multi-walled carbon nanotubes [20] and Au vertical hematite nanotube arrays [21] are interfered by ethanol that is omnipresent in hospitals and gyms from disinfectants (>100 ppm [22]).

Recently, a packed bed catalytic filter of flame-made ZnO nanoparticles at 260 °C was introduced that removed ethanol selectively (i.e., up to 185 ppm) over acetone [23] by exploiting the ZnO’s distinct surface basicity [24]. However, this filter did not remove critical methanol, isoprene and H_2_. While Co_3_O_4_ and PdO nanocatalysts on In_2_O_3_ hollow spheres removed toluene, CO, H_2_ and NH_3_ quite effectively over acetone, their performance on ethanol has not been evaluated yet to assess possible interference [25]. This was addressed with a 0.2 mol% Pt/Al_2_O_3_ catalyst at 135 °C that featured unprecedented acetone selectivity (>250) over ethanol, H_2_, CO, isoprene, NH_3_, methanol, formaldehyde, acetaldehyde, toluene and m-xylene at 90% relative humidity (RH) [19], as proven also for human breath with mass spectrometry [26]. The high acetone selectivity was associated [19] with interferant oxidation (e.g., ethanol/methanol [27]) by hydroxyl-related species on Al_2_O_3_ surfaces. In contrast, acetone remains intact since the Lewis acidic sites required for acetone conversion [28] are blocked by dissociating water [29] present in highly humid breath. The addition of 0.2 mol% Pt enhanced the catalytic conversion to lower temperatures. However, a consistent drawback of these catalytic packed bed filters remained their elevated operation temperatures (e.g., 135 °C [19]), requiring additional heating power. This impedes their integration into hand-held, battery-driven devices or even wearables that are desirable for mobile health care [30].

The reactivity of catalysts, and thus operational temperature, is influenced strongly by noble metals, as they lower the activation energy, for instance, by dissociatively adsorbing highly reactive oxygen [31]. Specifically for Pt, cluster size, oxidation state [32], loading [33] and dispersion [34] determine its reactivity. For instance, metallic Pt clusters of 6 nm size showed higher reactivity to methanol, another endogenous breath compound [35], than smaller (i.e., <1 nm) and oxidized PtO_x_ clusters [32]. Similarly, ethanol reactivity was increased by an order of magnitude when increasing Pt cluster size from 2.1 to 7.4 nm [36] as was the case also for methane and cyclopentane combustion with increasing Pt cluster sizes from 1.3 to 5.7 nm [37]. Finally, the loading and dispersion of noble metals on metal-oxide surfaces leads to a shift of conversion to lower temperatures, as was reported for toluene and propene combustion on 0.5–1.5 wt% Pt/Al_2_O_3_, while preserving selectivity [33].

Here, room-temperature catalytic filters are introduced that remove alcohols, aldehydes and inorganics (e.g., H_2_ and CO) selectively over acetone. This is achieved by flame spray pyrolysis (FSP) yielding highly dispersed Pt/PtO_x_ clusters (0–10 mol%) on Al_2_O_3_ nanoparticles at controlled composition [38]. Their crystal structure, cluster size distribution and specific surface area (SSA) were characterized by X-ray diffraction (XRD), electron microscopy and N_2_ adsorption. The catalytic performance towards methanol, ethanol (up to 100 ppm), isoprene and acetone was investigated at breath analysis-relevant 90% RH by proton-transfer-reaction time-of-flight mass spectrometry (PTR-ToF-MS). To demonstrate the filters’ immediate practical impact, it was connected to a flame-made, chemoresistive Si/WO_3_ sensor [39] and tested for selective acetone sensing.

## 2. Materials and Methods

### 2.1. Pt/Al_2_O_3_ Fabrication

Pt/Al_2_O_3_ nanoparticles were prepared by FSP [38] with a reactor described elaborately elsewhere [40]. Therefore, Pt acetylacetonate (Alfa Aesar, Pt ≥ 48.0%) and Al-tri-sec-butoxide (Sigma-Aldrich, 97%) were dissolved in a xylene/acetonitrile mixture (7:3 *v*/*v*) to achieve a total metal content (Pt + Al) of 0.5 M and a Pt loading of 0, 1, 3, 5 and 10 mol%. This corresponds in the product (Pt on Al_2_O_3_) to 0, 3.7, 7.2, 16.8 and 29.8 wt%, respectively. The precursor was fed at 5 mL/min through a nozzle and dispersed by 5 L/min oxygen at a pressure drop of 1.7 bar. A ring-shaped premixed methane/oxygen flow (1.25 and 3.2 L/min, respectively) ignited the spray and sustained the flame. The powder was collected with a vacuum pump (Seco SV 1025 C, Busch) on a water-cooled glass fiber filter (GF6 Albert-Hahnemuehle, D = 257 mm) at 50 cm height above the burner. The particles were scraped off the filter with a spatula and thermally stabilized by annealing in air at 500 °C for 1 h in an oven (Carbolite Gero GmbH, 30–3000 °C, Neuhausen, Germany).

### 2.2. Pt/Al_2_O_3_ Characterization

X-ray diffraction (XRD) patterns were recorded at 2*θ* = 15–70° with a scanning step and speed of 0.011° and 0.0057°/s using a Bruker AXS D8 Advance diffractometer operated at 40 kV and 30 mA. Crystal phases were identified by the software Bruker Diffrac.eva V3.1 by comparison to structural parameters of cubic γ-Al_2_O_3_ (PDF 10–0425), cubic Pt (PDF 01-1311) and tetragonal PtO (PDF 85-0714). To identify peak shifts, crystalline NiO (Bunsenite, ~325 mesh, Sigma-Aldrich, Buchs, Switzerland, PDF 47-1049) was admixed in a 1:1 w/w ratio as internal standard [41] to align the XRD patterns. The crystallite sizes were calculated using the Scherrer equation:

$$D=\frac{K\lambda }{\beta \cos\theta }$$

where *D* is the crystallite size in nm; *K* is the Scherrer constant (i.e., 0.9); *λ* is the wavelength of the CuK_α_ X-ray source (i.e., 0.15406 nm); *β* is the full width at half maximum (FWHM) and *θ* is the peak position (both in radian). Bimodality of Pt was identified by subtracting first the pure Al_2_O_3_ pattern and subsequent peak deconvolution at 2*θ* = 39.7° using the software OriginPro 2018G (OriginLab Corporation). Al_2_O_3_ crystal sizes were determined with the Scherrer Equation at 2*θ* = 67.1°, while for 10 mol% Pt, the Al_2_O_3_ peak was first deconvoluted from the Pt peak.

For in situ XRD analysis during H_2_ reduction, a high temperature cell was used (HTK 1200N, Anton Paar, Graz, Austria). For this, the 3 mol% Pt/Al_2_O_3_ powder was filled into a 16 mm sampler holder featuring a 1 mm high edge (Anton Paar, Graz, Austria). The particles were flushed with a constant flow of 50 mL/min H_2_/Ar and the temperature was increased stepwise with 0.5 °C/s from 30 °C to 150, 250 and 350 °C. The particles remained in isothermal conditions for 2 h at each temperature prior to analysis.

N_2_ adsorption was carried out at 77 K using a Tri-Star Micromeritics II Plus. All powders were degassed at 150 °C in N_2_ for 1 h prior to the analysis to remove humidity and other residues from the particle surfaces. The SSA was determined with the Brunauer-Emmett-Teller (BET) model. Particle sizes were calculated assuming separate Al_2_O_3_ and Pt spherical particles and an averaged density based on the relative amounts of Al_2_O_3_ (3.95 g/cm^3^) and Pt (21.45 g/cm^3^) while neglecting PtO_x_.

For electron microscopy imaging, the particles were dispersed in ethanol and deposited onto a copper grid-supported perforated carbon foil. High resolution transmission electron microscopy (HRTEM) images were acquired on a JEM-ARM300F (GrandArm, JEOL, Tokyo, Japan) operated at 300 kV. Furthermore, a high-angle annular dark-field scanning transmission electron microscope (HAADF-STEM, HD-2700CS, Hitachi, Tokyo, Japan) operated at 200 kV and equipped with an Energy-dispersive X-ray spectroscopy detector (EDXS) was used to image the Pt/Al_2_O_3_ particles. The area-derived particle diameters were determined from HAADF-STEM images using the NanoDefine tool of ImageJ (version 1.53c) to measure the Pt particle/cluster area and assuming spherical particles. A lognormal fit was applied to derive the number count-based particle size distribution (PSD) and to identify the mean geometric diameter (d_g_) and standard deviation (σ_g_).

### 2.3. Catalytic Evaluation

The measurement setup comprised a gas mixing unit [42] that was connected to a catalytic reactor [23] through inert and heated Teflon tubing to mitigate water condensation and analyte adsorption. The gas mixing unit was composed of several high-resolution mass flow controllers (Bronkhorst) that dosed the calibrated analytes (i.e., ethanol (10 and 495 ppm), acetone (15 ppm), isoprene (15 ppm) and methanol (15 ppm), all from PanGas in synthetic air, into a hydrocarbon-free air stream (PanGas, C_n_H_m_ and NO_x_ ≤ 100 ppb) at 150 mL/min, unless otherwise specified. Humidity was admixed by guiding synthetic air through a 125 mL glass bubbler (Drechsel bottle, sintered glass frit, Sigma-Aldrich, Buchs, Switzerland) filled with ultrapure water (Milli-Q S90, Merck, Switzerland) and adjusted to reach 0–90% RH that remained quite stable (±0.3% [43]) during 12 h of continuous operation, as confirmed with a humidity sensor (SHT2x, Sensirion AG, Stäfa, Switzerland).

The catalyst consisted of 30 mg nanoparticles prepared as a packed bed inside a tailor-made quartz glass reactor (inner diameter = 4 mm) and fixated at both ends with quartz wool and quartz sand [23]. Only where specified, catalysts were reduced with 5% H_2_/Ar (PanGas) at 350 °C for 2 h prior to measurements. The reactor was then placed inside an oven (Nabertherm, P320, Lilienthal, Germany) and heated to the desired temperature (i.e., 25–400 °C). Effluent gas at the filter outlet was analyzed using a PTR-ToF-MS 1000 (Ionicon, Innsbruck, Austria) [44] with H_3_O^+^ as precursor ions at 600 V drift voltage, 60 °C drift temperature and 2.4 mbar drift pressure. Analyte concentrations were identified at *m/z* values of 33.03 (methanol [45]), 47.05 (ethanol [46]), 59.05 (acetone [46]) and 69.07 (isoprene [45]). Prior to the measurements, 5-point calibrations were carried out with each analyte. Analyte conversion was calculated from the analyte (*i*) concentration at the inlet (*c_in_*_,*i*_) and outlet (*c_out_*_,*i*_) using the following formula:

$$Conversion=\left(1-\frac{c_{out,i}}{c_{in,i}}\right)$$

### 2.4. Detector Fabrication and Evaluation

Sensing tests were carried out with a flame-made 10 mol% Si-containing WO_3_ (Si/WO_3_) sensor [39]. The precursor solution consisted of ammonium metatungstate hydrate (Sigma-Aldrich, ≥97%) and hexamethyldisiloxane (Sigma-Aldrich, ≥98%) dissolved in a 1:1 (*v*/*v*) mixture of ethanol (Fluka, ≥99.8%) and diethylene glycol monobutyl ether (Sigma-Aldrich, ≥98%). For comparison, also a 0.5 mol% Pd/SnO_2_ sensor was prepared [47] with a precursor solution containing tin(II)-ethylhexanoate (STREM Chemicals, purity ≥ 90%) and palladium(II)-acetylacetonate (Sigma Aldrich, purity ≥ 99%) dissolved in xylene (Sigma Aldrich, ≥96%) with a total metal content of 0.5 M. The FSP conditions were identical to the above for Pt/Al_2_O_3_ particles. The Si/WO_3_ and Pd/SnO_2_ nanoparticles were directly deposited onto an Al_2_O_3_ substrate (15 × 13 × 0.8 mm^3^, Electronic Design Center, Case Western Reserve University) featuring interdigitated electrodes and a Pt back-heater [48], situated at 20 cm height above the burner (HAB). Subsequently, the substrate was lowered to 14 cm HAB and in situ annealed [49] with a particle-free xylene flame for 30 s. Thereafter, the sensor was annealed in air at 500 °C for 5 h (Carbolite Gero GmbH, Neuhausen, Germany) and mounted on a Macor holder installed inside a Teflon chamber [48]. There, it was heated to 350 °C (being the optimal temperature for acetone sensing [48]) by passing a DC current (R&S HMC8043) through the back-heater of the sensor substrate. The ohmic film resistance was recorded using a multimeter (Keithley, 2700).

Sensing tests were performed both with an inactive (i.e., pure Al_2_O_3_) and active (3 mol% Pt/Al_2_O_3_) catalytic filter (30 mg) that was fixated at both sides with quartz wool and quartz sand inside a compact glass tube (i.e., 4 mm inner diameter, 5 cm length, Supelco, Sigma Aldrich, Buchs, Switzerland). The filter was then connected downstream of the gas mixing unit and upstream of the sensor. Note that the filter was slightly heated (i.e., to 40 °C), being the standard in breath analysis [50]. Sensing tests were performed with the above analytes, and additionally with 2-propanol (250 ppm), acetaldehyde (15 ppm), CO (50 ppm), H_2_ (50 ppm, all PanGas, in synthetic air) and formaldehyde (10 ppm, in N_2_). The sensor response to each analyte was calculated as:

$$Response=\;\frac{R_{air}}{R_{analyte}}-1$$

where *R_air_* and *R_analyte_* represent the film resistances in air or during analyte exposure, respectively. The acetone selectivity was defined as the ratio between the acetone response and that to a specific analyte following IUPAC guidelines [51].

## 3. Results and Discussion

### 3.1. Tailoring Pt Size and Dispersion

**Figure 1:**
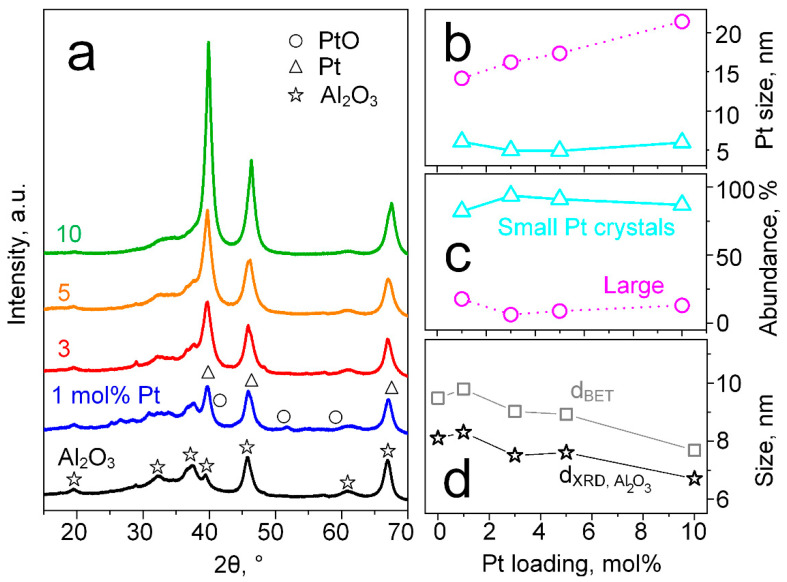
(**a**) XRD patterns of as-prepared pure (black), 1 (blue), 3 (red), 5 (orange) and 10 mol% (green) Pt/Al_2_O_3_ particles with reference peaks for cubic Al_2_O_3_ (stars), Pt (triangles) and PtO (circles). Note that all spectra were normalized to the Al_2_O_3_ peak at 2*θ* = 67.1°, that is less affected by Pt content. (**b**) Average crystallite size and (**c**) relative abundance of small (triangles) and larger (circles) Pt crystals, respectively, as a function of Pt content. Bimodal Pt crystal sizes were calculated using the Scherrer Equation after deconvolution of the Pt peak at 2*θ* = 39.7° (Figure S1). The abundance was determined from the respective peak areas. (**d**) Pt/Al_2_O_3_ particles size (BET-equivalent, squares) as determined by N_2_ adsorption and Al_2_O_3_ crystal size (stars) as calculated with the Scherrer Equation at 2*θ* = 67.1° as a function of Pt content.

First, the crystallinity and crystal sizes of flame-made and annealed (500 °C for 1 h) powders with 0–10 mol% Pt on Al_2_O_3_ were investigated. In the absence of Pt, flame-made Al_2_O_3_ forms the cubic γ-phase (stars, Figure 1a), in agreement with the literature [38]. Most importantly, adding up to 10 mol% Pt systematically emerges the peaks at 2*θ* = 39.7° and 46.3°. This suggests the formation of highly crystalline and metallic Pt crystals with peaks (triangles, Figure 1a) that overlap with those of γ-Al_2_O_3_, as had been observed previously for wet-impregnated Pt/Al_2_O_3_ after rather similar (500 °C for 2 h) annealing [52]. Importantly, no crystalline PtO (circles) is detected, which is desired for highly reactive catalytic filters [32]. While it is known that metallic Pt dominates acidic supports [53] like Al_2_O_3_ [24], the presence of some amorphous PtO_x_ (not detectable by XRD) has been revealed with an extended X-ray absorption fine structure (EXAFS) on flame-made and similarly annealed Pt/Al_2_O_3_ (i.e., 2 h at 500 °C) before [54].

Interestingly, the Pt peaks feature sharp tips and broader bases, indicative of bimodal crystal size distributions [55]. In fact, deconvolution of the peak at 2*θ* = 39.7° (Figure S1) reveals smaller and larger Pt crystals. The smaller Pt crystals (triangles, Figure 1b) feature rather constant sizes of 5.5 ± 0.6 nm, close to the 6 nm and 7.4 nm, that showed high reactivity towards methanol [32] and ethanol [36]. Such small Pt crystals dominate (relative abundance 82–94%, Figure 1c) for all Pt contents over larger ones (with sizes ranging from 14.2 to 21.4 nm, circles in Figure 1b) and are probably stabilized by strong anchoring on penta-coordinated Al^3+^ sites on the γ-Al_2_O_3_ (100) surfaces [56], as had been reported for Pt/Al_2_O_3_ before [57].

It is noteworthy that the addition of Pt decreased the γ-Al_2_O_3_ crystal size from 8.1 to 6.7 nm (Figure 1d, stars). This might indicate some (substitutional or interstitial) incorporation of Pt into the Al_2_O_3_ lattice, that was investigated further by XRD peak shift analysis (Figure S2) with an internal standard (i.e., crystalline NiO [41]). However, no lattice distortion was observed, suggesting no Pt incorporation, which is likely due to the significantly larger ionic radii of Pt (i.e., 80 pm Pt^2+^ or 63 ppm Pt^4+^ [58]) compared to Al (54 pm Al^3+^ [58]) at a coordination number of VI, as relevant for γ-Al_2_O_3_. Note that the BET-equivalent particle diameters (determined by N_2_ adsorption) for 0–10 mol% Pt were 7.7–9.5 nm (Figure 1d, squares), consistently larger than the γ-Al_2_O_3_ crystal size (stars), suggesting some polycrystallinity.

**Figure 2:**
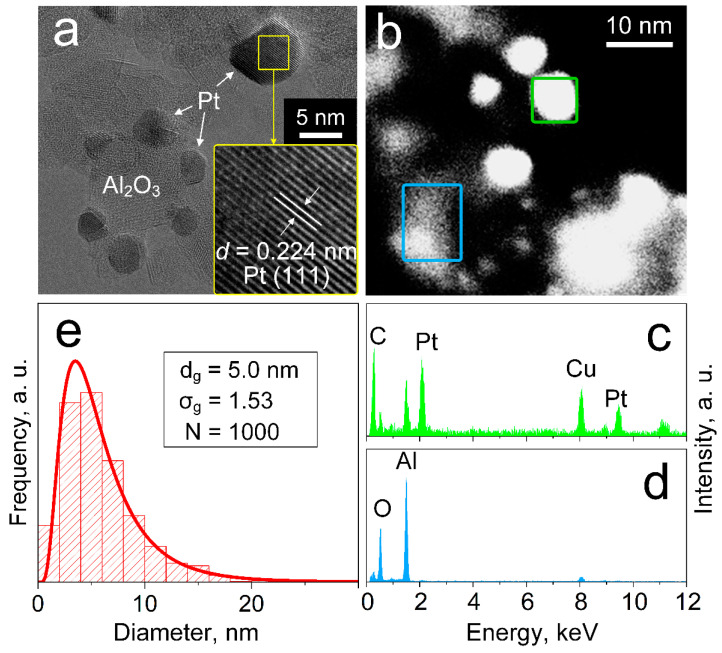
(**a**) HRTEM image of 3 mol% Pt/Al_2_O_3_. The inset shows the lattice fringes corresponding to the Pt (111) crystal plane. (**b**) HAADF-STEM image of such Pt/Al_2_O_3_ particles. EDXS analysis of the green (**c**) and blue (**d**) areas in (**b**). (**e**) Particle size distribution as determined from HAADF-STEM, together with the mean geometric diameter (d_g_), standard deviation (σ_g_) and number (N) of counted particles.

The morphology and dispersion of the Pt crystals was investigated further by electron microscopy, exemplarily for 3 mol% Pt/Al_2_O_3_. HRTEM reveals the presence of separate Al_2_O_3_ (brighter) and Pt (dark) particles/clusters (Figure 2a) that both feature a rather spherical shape. Their faceted appearance and visible lattice fringes support high crystallinity, in line with XRD (Figure 1a). When magnifying such a Pt particle/cluster (inset of Figure 2a), a lattice spacing of 0.224 nm is measured that matches well with the Pt (111) plane. Most such Pt crystals seem well dispersed over the Al_2_O_3_ support, forming fine surface clusters that are favorable for catalytic filtering given their large reactive surface areas.

A further distinction between Pt and Al_2_O_3_ particles/clusters is provided by HAADF-STEM, where the Pt particles appear now brighter than Al_2_O_3_ due to their higher scattering potential (Figure 2b). In fact, corresponding EDXS analysis validates the presence of mostly Pt (Figure 2c, green square in Figure 2b) for bright clusters while Al and O (Figure 2d, blue square in Figure 2b) dominate the darker particles. Note that the C and Cu signals originate from the sample grid (i.e., perforated carbon foil on Cu grid, see Materials and Methods).

The size distribution (Figure 2e) for 1000 Pt particles/clusters was determined from such HAADF-STEM images (Figure S3). A lognormal fit (red line) yields a geometric average diameter (d_g_) and standard deviation (σ_g_) of 5 nm and 1.53, respectively, that agrees well with the average crystal size of the small Pt clusters (Figure 1b: 5 nm). Note that no bimodality is visible in the number frequency size distribution here, likely since the relative abundance of larger particles is rather small (Figure 1c). Remarkably, quite similar d_g_ (i.e., 5–5.9 nm) are obtained for all Pt contents (Figure S4). This should be associated with the aforementioned strong anchoring of the small Pt clusters on the Al_2_O_3_ [56] that prevents their sintering during annealing, while the larger clusters grow with increasing Pt content (Figure 1b). As a result, Pt content affects primarily the surface loading, while the size of small Pt clusters and their dispersion remain rather invariant.

### 3.2. Catalytic Reactivity

**Figure 3:**
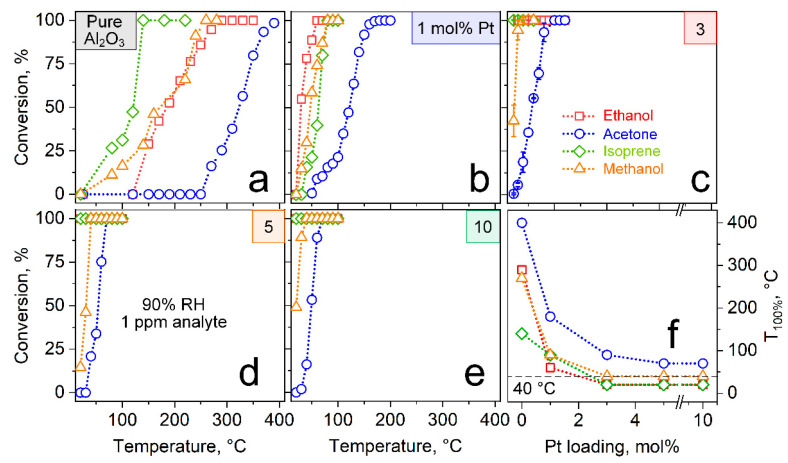
Conversion of 1 ppm ethanol (squares), acetone (circles), isoprene (diamonds) and methanol (triangles) over pure Al_2_O_3_ (**a**) and with 1 (**b**), 3 (**c**), 5 (**d**) and 10 mol% (**e**) Pt at 90% RH, as determined by PTR-ToF-MS. Error bars in (**c**) indicate the standard deviations for three identically prepared packed beds. (**f**) The corresponding temperature of complete conversion (T_100%_) as a function of Pt loading. Minimum required filter temperature (i.e., 40 °C) to avoid water condensation in breath analysis is indicated as horizontal dashed line.

The catalytic performance of these nanoparticles was tested by analyzing the exhaust of a 30 mg Pt/Al_2_O_3_ packed bed with bench-top PTR-ToF-MS (Figure 3). Tests were performed with 1 ppm of gaseous acetone (circles), isoprene (diamonds), methanol (triangles) and ethanol (squares) at 90% RH to simulate breath-realistic conditions. When increasing the temperature sequentially from 25–400 °C, the pure Al_2_O_3_ catalyst (Figure 3a) converts first isoprene (100% conversion at 140 °C) followed by methanol (260 °C) and ethanol (290 °C). Remarkably, acetone starts to convert only at 270 °C and complete conversion is observed even after 390 °C, resulting in distinct acetone selectivity, as had been shown previously for ethanol and acetone [19].

Most importantly, when increasing the Pt content, the conversion curves are systematically shifted towards lower temperatures (Figure 3b–e). Specifically, all interferants are converted completely at 90 °C with only 1 mol% Pt and this is further reduced to 40 °C in the case of 3, 5 and 10 mol% Pt. Note that the filter should not be operated below 40 °C, which is standard [35,59] in breath analysis to avoid water condensation from rather humid exhalations (i.e., >90% RH [60] at body temperature). The high reactivity of 3–10 mol% Pt/Al_2_O_3_ at room temperature should be attributed to the well dispersed Pt clusters of 5.0–5.9 nm size (Figure 2 and Figure S4). In fact, similar Pt cluster sizes were reported to be highly reactive for methanol [32], ethanol [36] and hydrocarbons [37], as had been specified in the Introduction.

Most importantly, the high acetone selectivity is maintained for all Pt contents, as the acetone is converted consistently at higher temperatures (Figure 3f, circles) than the confounders. For instance, for three identically prepared 3 mol% Pt/Al_2_O_3_ packed beds at 40 °C, only 18.7% ± 5.8% (Figure 3c, circles) of the acetone are lost while all confounders are removed completely. To further investigate this acetone selectivity, we reduced the 1 and 10 mol% Pt/Al_2_O_3_ in H_2_ prior to catalytic characterization (Figure S5). While this resulted in even lower conversion temperatures for all confounders, the acetone selectivity was deteriorated (i.e., 50.6 and 59.3% acetone conversion at complete interferant removal for 1 and 10 mol% Pt, respectively). This suggests the presence of less reactive [61] but apparently more acetone-selective PtO_x_ on the metallic Pt clusters [54], that might be amorphous since it is not detectable by XRD (Figure 1). In fact, in situ XRD (Figure S6) during this treatment also revealed neither changes of the crystalline phases nor their sizes. However, the detailed reaction mechanism remains to be clarified.

**Figure 4:**
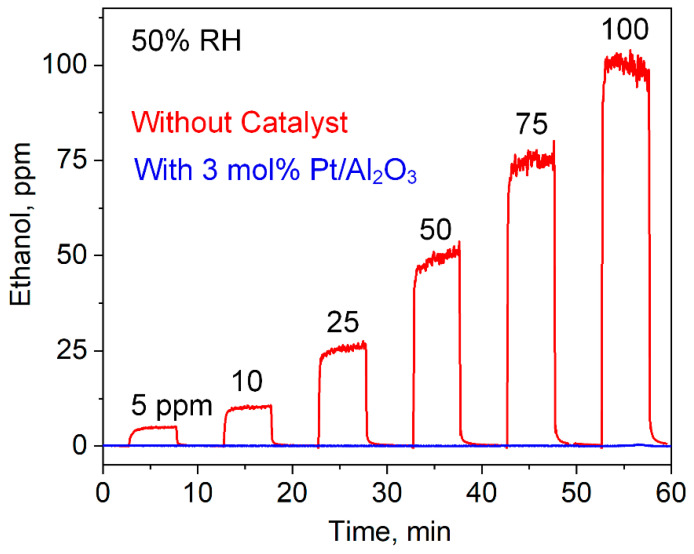
Exposure to 5–100 ppm ethanol, as detected by the PTR-ToF-MS without (red) and with (blue) the 3 mol% Pt/Al_2_O_3_ filter at 40 °C. Note that 50% RH is used instead of 90% due to a limitation of the measurement setup at such high analyte concentrations.

To challenge the catalytic filter further, the 3 mol% Pt/Al_2_O_3_ packed bed at 40 °C was tested for the removal of 5–100 ppm ethanol (Figure 4) at 50% RH. Such high ethanol concentrations can be present in hospitals from sanitizers [22] and are removed by the catalytic filter completely (red vs. blue line), as confirmed by PTR-ToF-MS. This is, at least, competitive to filters based on Au/Fe_2_O_3_ (at 200 °C) [62] and ZnO (at 260 °C) [23], that had to be heated though. Furthermore, the catalyst was fairly robust to changing RH between 30 and 90% RH (acetone loss 46–14% at complete interferant conversion, Figure S7a), as it is usually present in room air and exhaled breath, and performs well also for other flows (i.e., 50–200 mL/min, Figure S7b) through the packed bed.

### 3.3. Selective Acetone Sensing with Room Temperature Filter

**Figure 5:**
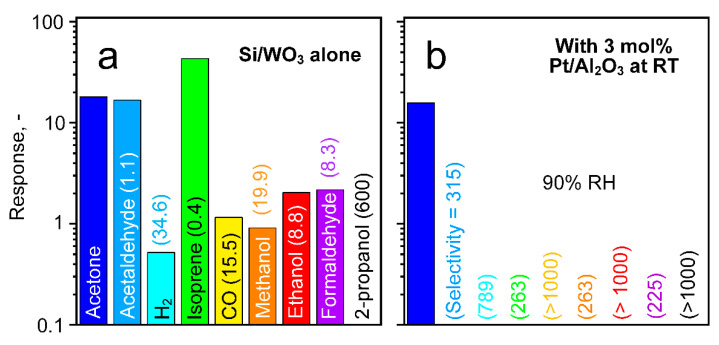
Response of a Si/WO_3_ sensor to 1 ppm acetone, acetaldehyde, H_2_, isoprene, CO, methanol, ethanol, formaldehyde and 2-propanol (**a**) with 30 mg pure Al_2_O_3_ (i.e., inactive, Figure 3a) and (**b**) 3 mol% Pt/Al_2_O_3_ (i.e., active, Figure 3c) at 40 °C and 90% RH. Acetone selectivity is shown in parentheses. Note the logarithmic ordinate scale.

To demonstrate immediate practical impact, 30 mg of such 3 mol% Pt/Al_2_O_3_ at 40 °C were placed as packed bed filter ahead of a flame-made, chemoresistive Si/WO_3_ [48] sensor. When testing the sensor alone to 1 ppm acetone and eight breath-relevant interferants at 90% RH (Figure 5a), it responded to acetone (18) but showed an even higher response to isoprene (43.2) and was interfered by ethanol (2) and H_2_ (0.5) that can be present at orders of magnitude higher concentrations than acetone. The resulting selectivity at the same analyte concentrations range from 0.4–600 and are in fair agreement with earlier reports for ethanol (6.7 but at 400 °C [63]) and isoprene (0.5 [19]). However, these are insufficient and can lead to significant measurement errors, for instance, when monitoring breath acetone in situ during cardio-respiratory fitness-adapted [64] cycling [26].

This is eliminated effectively by the filter. In fact, the 3 mol% Pt/Al_2_O_3_ packed bed at 40 °C reduces these interferences (Figure 5b). Now, only acetone is detected with a response of 15.8, while the interferants are hardly recognized anymore (responses <0.1), in line with the catalytic characterization (Figure 3c). This results in high selectivity for all analytes (≥225, Figure 5b in brackets), and is highest for CO, ethanol and 2-propanol (all >1′000). Note that the acetone response reduction of 12.2% (Figure 5b) is in fair agreement with Figure 3c (18.7 ± 5.8%). The obtained selectivities are comparable to the ones achieved with a 0.2 mol% Pt/Al_2_O_3_ filter (at 135 °C); however, operated here at room temperature. Moreover, it outperforms state-of-the-art acetone sensors (e.g., Al-ZnO [18], Si/WO_3_ [19], Co-doped ZnO nanofibers [15], TiO_2_/WO_3_ nanocrystals [16], SnO_2_ with multi-walled carbon nanotubes [20] and Au vertical hematite nanotube arrays [21]).

**Figure 6:**
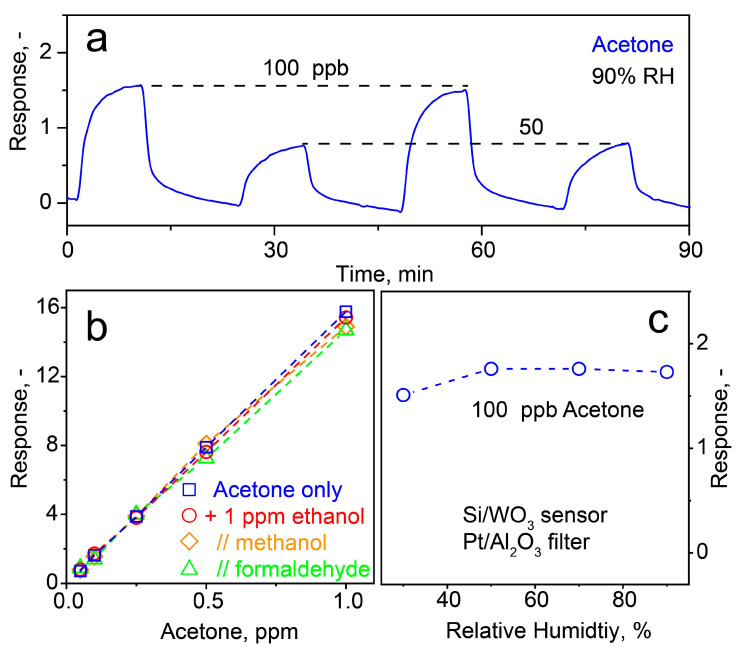
(**a**) Detector (i.e., Si/WO_3_ sensor with Pt/Al_2_O_3_ filter at 40 °C) response to consecutive exposures to 100 and 50 ppb acetone at 90% RH. (**b**) Detector response to 0.05, 0.1, 0.25, 0.5 and 1 ppm acetone as single analyte (squares) and in mixture with 1 ppm ethanol (circles), methanol (diamonds) and formaldehyde (triangles) at 90% RH. (**c**) Detector response to 100 ppb acetone at 30–90% RH.

End-tidal breath acetone levels are usually between 148–2744 ppb, as observed during weekly breath tests of 30 volunteers during 6 months [59]. Therefore, the detector was exposed subsequently to 100 and 50 ppb of acetone (Figure 6a). These concentrations are clearly distinguished with high signal to noise ratios (i.e., SNR > 50). Note that the extrapolated LOD (at SNR = 3) is even 2 ppb. Importantly, the detector features also a good repeatability (dashed lines, Figure 6a) with a response change <5% and excellent reproducibility of ±5.8% for the filter (error bars in Figure 3c at 40 °C) and 8% [19] for the Si/WO_3_ sensor alone, when testing three identically prepared samples.

Since exhaled human breath is a mixture of analytes, we tested also binary combinations of these acetone concentrations with 1 ppm of ethanol (circles, Figure 6b), methanol (diamonds) and formaldehyde (triangles). Most importantly, the detector response to acetone hardly changes (e.g., 15.2 ± 0.5 at 1 ppm), highlighting its excellent selectivity. Finally, the detector was tested for its RH robustness when sensing 100 ppb acetone (Figure 6c). Remarkably, the response changed only little from 1.5 to 1.7 between 30–90% RH, demonstrating outstanding humidity robustness. Previous studies [63] showed reduced acetone response at increasing RH for the Si/WO_3_ sensor alone that apparently compensates for the filter’s higher acetone loss (Figure S7a).

## 4. Conclusions

We demonstrated the systematic design of a room temperature catalytic filter for selective acetone sensing by optimizing the cluster size and loading of Pt/PtO_x_ on Al_2_O_3_ nanoparticles with FSP. Such Pt/PtO_x_ clusters were rather dispersed on the Al_2_O_3_ and showed bimodal distribution. Small Pt clusters were predominantly present and their size remained rather constant (d_g_ = 5.0–5.9 nm) when altering Pt content, probably by stabilization on penta-coordinated Al^3+^ sites. Catalytic filters of such 3–10 mol% Pt/Al_2_O_3_ nanoparticles were highly reactive already at 40 °C, as confirmed by the complete conversion of up to 100 ppm ethanol with high robustness to 50–90% RH. We also suggested the presence of some PtO_x_, that was less reactive than metallic Pt but seemed beneficial for acetone selectivity.

As a proof-of-concept, such filters enhanced the acetone selectivity dramatically (i.e., ≥225 for eight critical confounders) that is required for accurate breath monitoring of lipolysis (e.g., during exercise and dieting). Due to the filter’s modular design, it can be combined flexibly also with other chemo-resistive sensors, like established [65] SnO_2_-based sensors (e.g., 0.5 mol% Pd/SnO_2_ [47], Figure S8), to turn them acetone-selective. Furthermore, it should even be compatible with different sensor types (e.g., electrochemical, optical, etc.). Importantly, the small filter size (i.e., 30 mg powder, 1.5 cm length × 0.4 cm diameter) allows its integration into compact and portable detectors. Since it requires no heating, it can be used readily with hand-held, smartphone-assisted and battery-driven devices [66] for breath acetone monitoring in mobile health care applications [67].

## Data Availability

Data are contained within the article or supplementary material. The data presented in this study are available in [insert article or supplementary material here].

## Supplementary Materials

The following are available online at https://www.mdpi.com/article/10.3390/ma14081839/s1, Figure S1: Deconvolution of Pt XRD peak, Figure S2: Pt/Al_2_O_3_ XRD patterns with NiO internal standard, Figure S3: STEM Pt cluster size determination, Figure S4: Particle size distributions, Figure S5: Catalytic performance before and after H_2_ reduction, Figure S6: In situ XRD during H_2_ reduction, Figure S7: Effect of relative humidity and flow. Figure S8: Filter performance with Pd/SnO_2_ sensor.
