# Supplementary material for: Room-Temperature Catalyst Enables Selective Acetone Sensing
Source: Materials (Basel). 2021 Apr 8;14(8):1839. doi: 10.3390/ma14081839 (PMC8067997; doi:10.3390/ma14081839)
Supplement: Supplementary file 1 [file materials-14-01839-s001.pdf]

materials

MDPI

Supplementary Materials

Room-Temperature Catalyst Enables Selective Acetone Sensing

Ines C. Weber [1], Chang-ting Wang [1] and Andreas T. Güntner [1,2,*]

[1] Particle Technology Laboratory, Department of Mechanical and Process Engineering, ETH Zurich, CH-8092 Zurich, Switzerland; iweber@ethz.ch (I.C.W.); chwang@student.ethz.ch (C.-t.W.)
[2] Department of Endocrinology, Diabetology, and Clinical Nutrition, University Hospital Zurich (USZ) and University of Zurich (UZH), CH-8091 Zurich, Switzerland
* Correspondence: andreas.guentner@ptl.mavt.ethz.ch; Tel.: +41-044-632-7411

Abstract: Catalytic packed bed filters ahead of gas sensors can drastically improve their selectivity, a key challenge in medical, food and environmental applications. Yet, such filters require high operation temperatures (usually some hundreds °C) impeding their integration into low-power (e.g., battery-driven) devices. Here, we reveal room-temperature catalytic filters that facilitate highly selective acetone sensing, a breath marker for body fat burn monitoring. Varying the Pt content between 0–10 mol% during flame spray pyrolysis resulted in Al_2O_3 nanoparticles decorated with Pt/PtO_x clusters with predominantly 5–6 nm size, as revealed by X-ray diffraction and electron microscopy. Most importantly, Pt contents above 3 mol% removed up to 100 ppm methanol, isoprene and ethanol completely already at 40 °C and high relative humidity, while acetone was mostly preserved, as confirmed by mass spectrometry. When combined with an inexpensive, chemo-resistive sensor of flame-made Si/WO_3, acetone was detected with high selectivity (≥225) over these interferants next to H_2, CO, form-/acetaldehyde and 2-propanol. Such catalytic filters do not require additional heating anymore, and thus are attractive for integration into mobile health care devices to monitor, for instance, lifestyle changes in gyms, hospitals or at home.

Keywords: nanotechnology; combustion synthesis; electronics; semiconductors; metal oxides; noble metals

Citation: Weber, I.C.; Wang, C.-t.; Güntner, A.T. Room-Temperature Catalyst Enables Selective Acetone Sensing. *Materials* **2021**, *14*, 1839. https://doi.org/10.3390/ma14081839

Academic editor: Antonio Gil Bravo

Received: 27 February 2021
Accepted: 1 April 2021
Published: 8 April 2021

Publisher's Note: MDPI stays neutral with regard to jurisdictional claims in published maps and institutional affiliations.

Copyright: © 2021 by the authors. Licensee MDPI, Basel, Switzerland. This article is an open access article distributed under the terms and conditions of the Creative Commons Attribution (CC BY) license (http://creativecommons.org/licenses/by/4.0/).

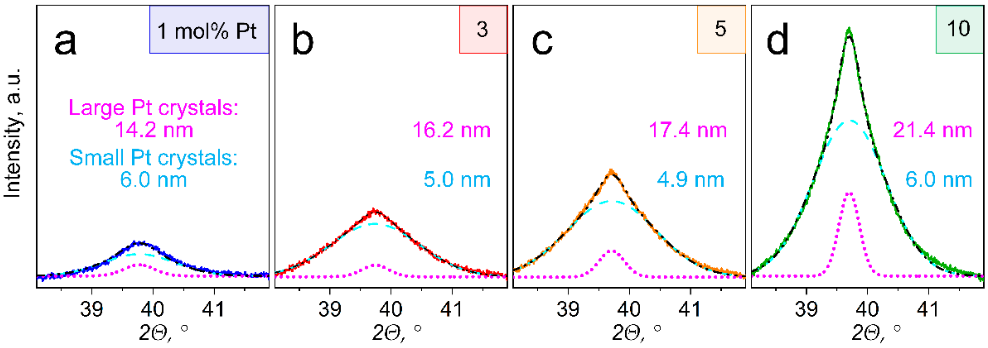

Figure S1. Peak deconvolution between 38° ≤ 2θ ≤ 42° for 1 (**a**), 3 (**b**), 5 (**c**) and 10 mol% Pt/Al_2O_3 (**d**). Small (turquoise, dashed) and larger (magenta, dotted) crystals are shown together with their superposition (black, dashed-dotted) that agrees well ($R^2 > 0.99$) with the XRD profiles. Crystal sizes were calculated using the Scherrer Equation.

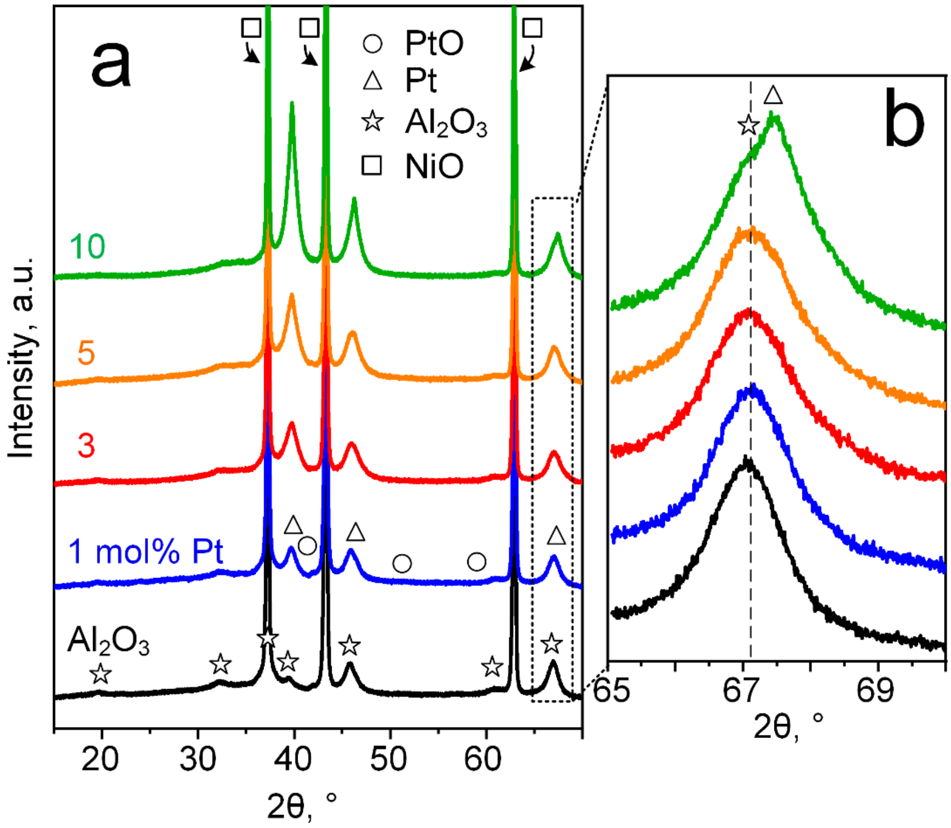

Figure S2. (**a**) XRD patterns of as-prepared pure (black), 1 (blue), 3 (red), 5 (orange) and 10 mol% (green) Pt/Al_2O_3 particles with additional 50 wt% NiO (squares) as internal standard. The reference peaks for Al_2O_3 (stars), Pt (triangles) and PtO (circles) are indicated. (**b**) Magnification of 2θ = 65–70° showing no peak shift with increasing Pt content. Dashed line indicates the main γ-Al_2O_3 peak at 2θ = 67.1°.

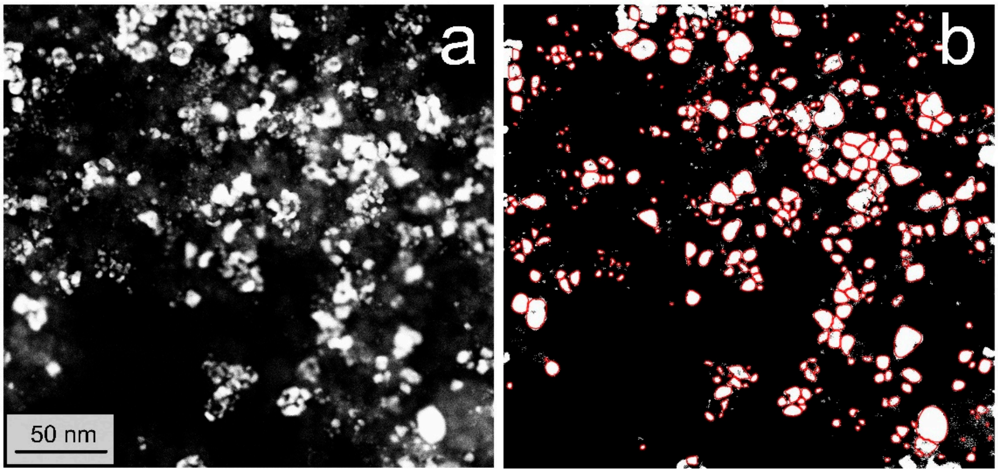

Figure S3. (**a**) HAADF-STEM image of 10 mol% Pt/Al_2O_3 exemplarily together with the marked areas (**b**), as determined by ImageJ (red lines) that were used to calculate the area-derived diameter assuming spherical particles.

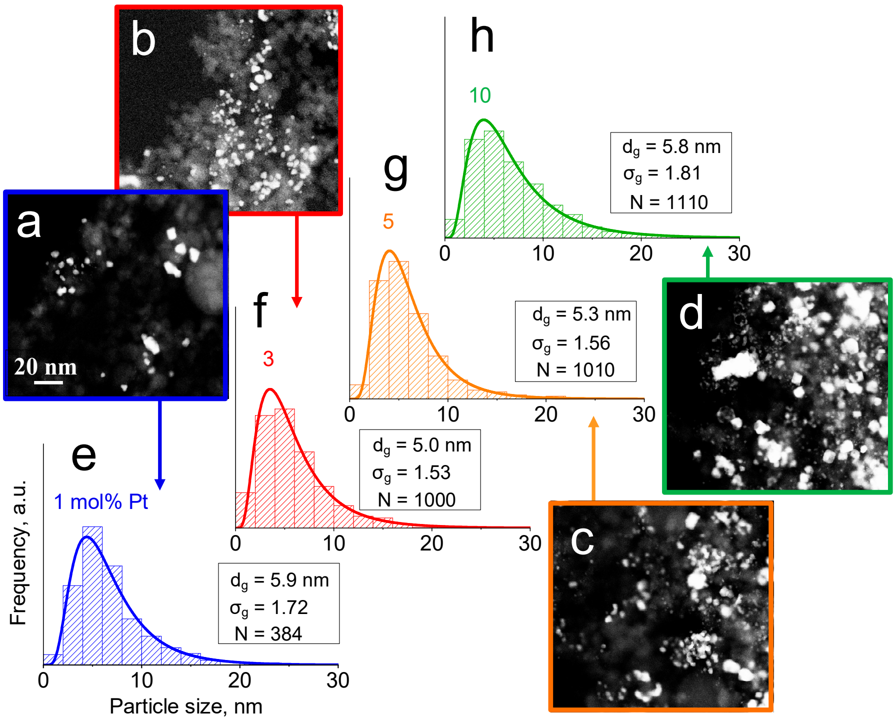

Figure S4. HAADF-STEM images of (**a**) 1, (**b**) 3, (**c**) 5 and (**d**) 10 mol% Pt/Al_2O_3 particles with their size distributions in (**e-h**), respectively. Lognormal fits are indicated together with the mean geometric diameter (d_g), standard deviation (σ_g) and number (N) of counted particles.

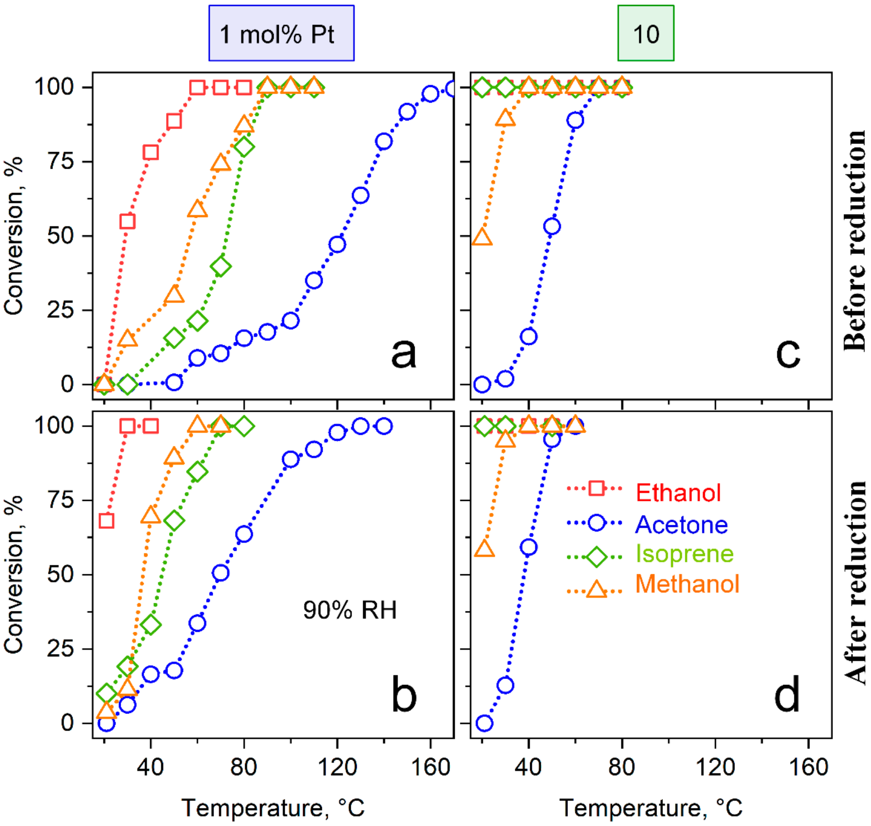

Figure S5. Catalytic conversion of 1 ppm acetone (circles), ethanol (squares), isoprene (diamonds) and methanol (triangles) at 90% RH over 1 (**a, b**) and 10 mol% Pt/Al_2O_3 (**c, d**) before (a, c) and after (b, d) particle reduction with 5% H_2/Ar at 350 °C for 2 h, as measured by PTR-ToF-MS. Note the different temperature scale to Figure 3b,e.

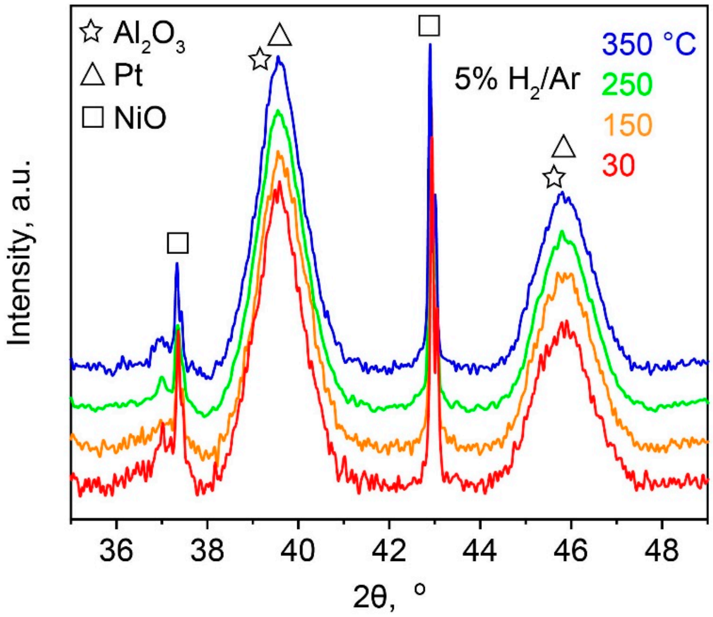

Figure S6. In situ XRD of 3 mol% Pt/Al_2O_3 measured in 5% H_2/Ar after heating for 2 h to 30 (red), 150 (orange), 250 (green) and 350 °C (blue) with reference peaks for cubic Al_2O_3 (stars) and Pt (triangles). Note that NiO (squares) served as an internal standard.

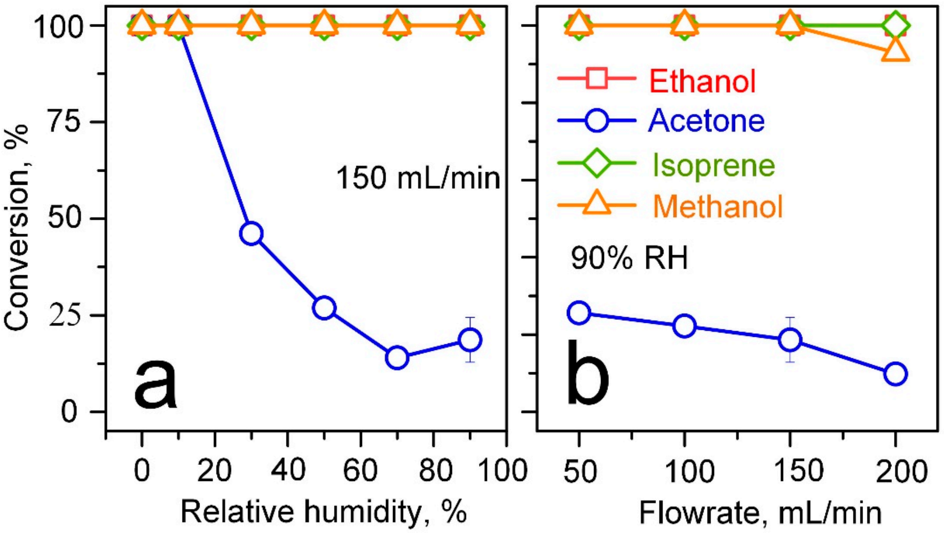

Figure S7. Catalytic conversion of 1 ppm acetone (circles), ethanol (squares), isoprene (diamonds), and methanol (triangles) over 3 mol% Pt/Al_2O_3 at 40 °C as a function of (**a**) RH and (**b**) flowrate. Error bars at 90% RH and 150 mL/min indicate the standard deviations for three identically prepared packed beds.

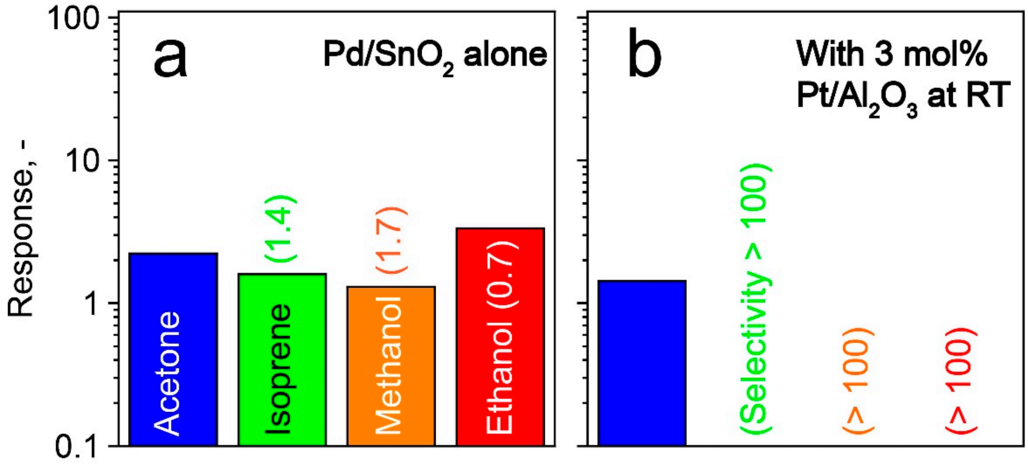

Figure S8. Response of a 0.5 mol% Pd/SnO_2 sensor at 350 °C to 1 ppm acetone, isoprene, methanol and ethanol with (**a**) 30 mg pure Al_2O_3 (i.e., inactive) and (**b**) 3 mol% Pt/Al_2O_3 (i.e., active) at 40 °C and 90% RH. Acetone selectivities are shown in parentheses. Note the logarithmic ordinate scale.
